# Natural selection contributes to food web stability

**DOI:** 10.1371/journal.pone.0227420

**Published:** 2020-01-10

**Authors:** Akihiko Mougi

**Affiliations:** Institute of Agricultural and Life Sciences, Academic Assembly, Shimane University, Matsue, Japan; Kyushu Institute of Technology, JAPAN

## Abstract

How biodiversity is maintained in ecosystems is a central issue in ecology. According to the evolutionary theory, heritable variations between individuals are important for the generation of species diversity, linking both intra and interspecific variations. The present food web model shows that intraspecific variations via natural selection also play crucial roles in maintaining the stability of large communities with diverse species. In particular, our computations indicate that larger communities need more intraspecific variation to be maintained and are powerfully stabilized when multiple traits are variable. Consequently, these variations are likely to be maintained in larger communities. Hence, intra and interspecific diversities may support each other during evolution.

## Introduction

Following the works of Charles Darwin, the disciplines of evolutionary biology and ecology have been directed at describing the processes by which biodiversity originates and is maintained [1,2]. Although the origin of biodiversity is generally understood in terms of Darwin’s theory of evolution [3–5], several theories of ecological coexistence have been proposed to describe the processes that maintain biodiversity [6–12]. Herein, I suggest that Darwinian evolution can explain the maintenance of biodiversity.

Evolution is a fundamental principle of the natural world, and variations in morphological, behavioral, and physiological traits between individuals within a species are essential requirements of evolution [13]. Because most traits exhibit some level of heritable variation, selection of fitter traits by environmental pressures leads to population changes that, in turn, alter the ecological properties of populations by influencing species interactions and community dynamics [14].

Recent studies on eco-evolutionary dynamics or the interplay of ecological and evolutionary processes on similar time scales have revealed reciprocal effects of ecological interactions on trait evolution and trait changes on ecological interactions, and consequent eco-evolutionary feedback systems have been posited [15–20]. Eco-evolutionary theories have also been used to understand the origins and evolutionary dynamics of biodiversity according to differences within and between species [5,21]. It also been used to describe the ecological processes that maintain biodiversity, particularly in terms of population dynamics of interacting species and the effects of these on ecosystem stability [14,22]. Yet, these theories describe simple communities with few species [23–25] and may fail to indicate how coevolution influences the stability of more complex communities [26–28].

It is expected that intraspecific and interspecific diversities interact through evolution. In the presence of intraspecific diversity in each species, coevolution can occur, leading to eco-evolutionary dynamics of the community [29,30]. In contrast, interspecific diversity can promote complexity of environments, leading to selective pressures for suitable and unsuitable phenotypes within a species, consistent with evolution due to natural selection [31]. Therefore, links between different levels of biodiversity are important in understanding how biodiversity is maintained [32–34]. Intraspecific diversity is lacking, particularly in models of more complex or species-rich communities [35]. Although some food web models of eco-evolutionary dynamics implicitly assume the presence of heritable variations [36,37], these models track mean population traits, leading to homogenous conspecifics at each time step. Even in studies that consider intraspecific variations, the variations were fixed [38] or were not controlled sufficiently to determine dose dependent effects on community dynamics [39]. Hence, it remains unclear how intraspecific variation itself, through evolution, affects the maintenance of more complex communities. Here I present an eco-evolutionary community dynamics model that explicitly includes varying degrees of heritable intraspecific variation. This model implicates intraspecific diversity as a natural selection process that contributes to the maintenance of community complexity.

## Methods

Consider a random food web with *N*_*S*_ species in which pairs of species *i* and *j* (*i*, *j* = 1,…, *N*_*S*_) are connected by a trophic interaction with a probability of *C* (connectance). Predator–prey roles are randomly assigned to two interacting pairs. Therefore, prey and predator are expected to have the same number. The maximum link number *L*_max_ is calculated as *N*_*S*_ (*N*_*S*_–1)/2. In the interaction matrix (one side or upper triangular matrix), the numbers of each sign of interactions (+ or–) are the product, *L*_max_(*C*/2), respectively. The number of the remaining part (0) is calculated as *L*_max_(1 –*C*). The random model can generate food webs with complex substructures common in real food webs [40–42]. In addition, I consider the cascade food web. In this model, for each pair of species *i*, *j* = 1,…, n with *i* < *j*, species *i* never consumes species *j*, whereas species *j* may consume species *i*. Therefore, it has the key features of real food webs on hierarchical structures among trophic levels [43].

The food web model is defined by the following ordinary differential equation:

$$\frac{dX_{i}}{dt}={\bar{w}}_{i}X_{i}$$ (1)

where *X*_*i*_ is the abundance of species *i*. In this equation, ${\bar{w}}_{i}$ (*i*∈1,…, *N*_*S*_) is the mean fitness of genotypes within the population of each species and is calculated as follows:

$${\bar{w}}_{i}=\sum_{j=1}^{N_{G}}f_{ij}w_{ij}$$ (2)

where *N*_*G*_ is the number of genotypes within the population of each species and *f*_*ij*_ (*i*∈1,…, *N*_*S*_) is the proportion of genotypes in the population of each species (Σ_*j*_ *f*_*ij*_ = 1). In this equation, *w*_*ij*_ represents the fitness of genotype *j* for each species, and is represented as follows:

$$w_{ij}=r_{ij}-s_{i}X_{i}+\sum_{k=1,k\neq i}^{N_{S}}\sum_{l=1}^{N_{G}}{a_{ijkl}f_{kl}X}_{k}$$ (3)

where *r*_*ij*_ (hereafter *r* in the text) is the intrinsic rate of change in a genotype of species *i*, *s*_*i*_ is the density-dependent self-regulation of species *i*, and *a*_*ijkl*_ is the interaction coefficient between a genotype of species *i* and species *k*. Interaction coefficients are defined as *a*_*ijkl*_ = *e*_*ijkl*_*α*_*ijkl*_ and *a*_*klij*_ = –*α*_*ijkl*_, where *α*_*ijkl*_ (hereafter *α* in the text) is the consumption rate and *e*_*ijkl*_ (<1) is the conversion efficiency. I used a non-zero self-regulation term in all species to avoid a confounding effect of an increase in interspecific links decreasing the number of heterotrophic species with no potential diet present in the web [44]. From the biological view, each species is either autotrophic or uses external resources. For simplicity, *e*_*ijkl*_ was set to a biologically feasible [45,46] constant value (*e* = 0.2) and *s*_*i*_ was set to a constant value (*s* = 1.0).

Intraspecific variation was defined by the differences in parameters among genotypes, *r* and *α*. The interaction links were common within each species (sharing same prey and predator species). Intraspecific interference competition was present among genotypes in each species. The common features between interaction links and intraspecific competition within species were considered to be species separation in this study. In contrast, I assumed no discrimination between intra and interspecific variations of genotypes on parameters *r* and *α*, i.e., genotype of the same species can considerably differ and be similar to the genotypes of different species [47,48]. However, this assumption will be relaxed by turning intra and/or interspecific variations.

To consider the dynamics of proportions of genotypes within populations of each modeled species, a replicator equation [49,50] was used as follows:

$$\frac{df_{ij}}{dt}=f_{ij}(w_{ij}-{\bar{w}}_{i}),$$ (4)

I did not explicitly consider the population dynamics of genotypes because the two models are essentially the same (S1 Text), and the difference is whether absolute or relative population size of genotypes is traced. It would be clear by substituting Eq (3) into Eqs (2) and (1). Then, Eq (1) is rearranged as

$$\frac{dX_{i}}{dt}=\sum_{j}\left(f_{ij}X_{i}\right)r_{ij}-s_{i}X_{i}^{2}+\sum_{j}\sum_{k}\sum_{l}a_{ijkl}\left(f_{ij}X_{i}\right)\left(f_{kl}X_{k}\right)$$ (5)

This clarifies that the growth term and interaction terms are weighted by the relative proportion of each genotype.

The differential Eqs (1) and (4) coupled the evolutionary and ecological dynamics of food webs. In each iterated simulation, initial species abundances and genotype frequencies were randomly chosen from the uniform distribution U[0, 1], and parameters *r* and *α* were randomly chosen from the uniform distributions $\bar{r}$U[0, 1] and $\bar{\alpha }$U[0, 1], where $\bar{r}$ and $\bar{\alpha }$ are constant parameters that control absolute intrinsic growth rates and interaction coefficients, respectively (these constant values are multiplied by random variables). In each simulation, I first made a stable food web, where all species coexisted. After the community converges to a stable coexistence equilibrium (at t = 10^3^) (the coexistence equilibrium is numerically confirmed to be locally stable based on the sign of real part of dominant eigenvalue of Jacobian Matrix), disturbance, randomly changing *r* and *α* ($\bar{r}$ = $\bar{\alpha }$ = 0.5) and genotype frequencies (disturbance is given in the same way as for the initial conditions) drives population fluctuation and evolution. In each simulation, I checked whether all species coexisted (*X*_*i*_ > 10^−5^ for all *i*) after a sufficient time period of t = 5 × 10^3^, which corresponded with the time taken for community persistence to reach an asymptote. These processes were repeated 500 times, and I measured community persistence [44] and the frequencies of all species co-occurring within all runs. This calculation was performed under each condition. For example, given a network type and levels of network properties, *C*, *N*_*S*_ and *N*_*G*_, interaction pairs, initial conditions (population sizes and proportions of genotype frequencies), and parameter values (*r* and *α*) are randomly determined in each simulation.

Two diversities, the species richness *N*_*S*_ and genotype number *N*_*G*_, are controlled within ranges, *N*_*S*_ (5–20) and *N*_*G*_ (1–5), respectively. *C* (0.2) has a fixed value in the main text. I also controlled the proportion of species with intraspecific variation within a community, *p*, which has > 2 genotypes (i.e. 1 –*p* species has 1 genotype).

## Results

**Fig 1:**
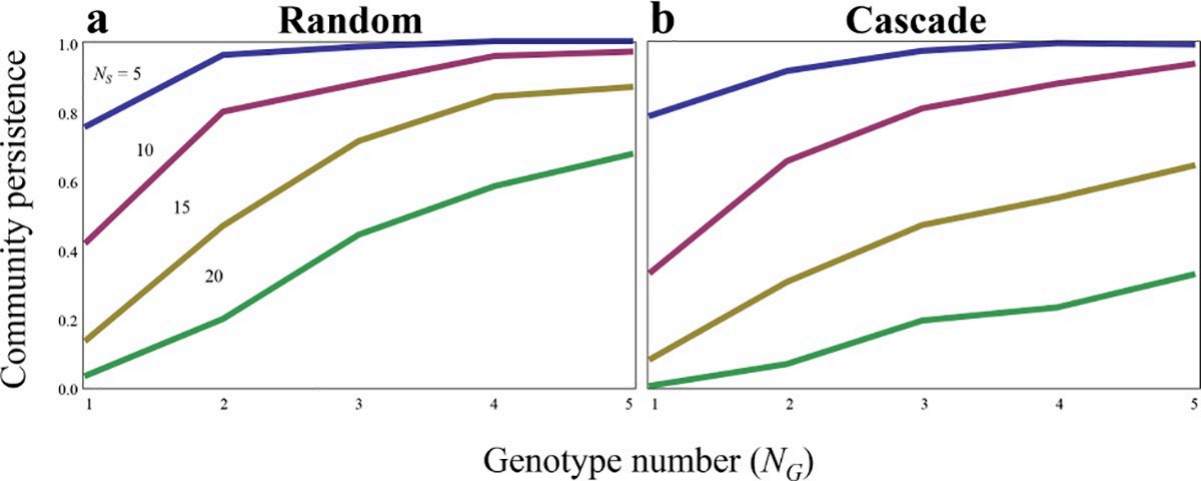
Effects of intra and interspecific diversity on stability. (a) Random food web (b) Cascade food web, *C =* 0.2.

Consider a typical ecological community model in which the food web comprises species with no intraspecific variations (*N*_*G*_ = 1). Congruent with earlier models, the present model suggests that it is difficult to maintain complexity in ecosystems, particularly in more complex or species-rich systems (Fig 1). Yet, evolution was favored by the introduction of intraspecific variations (*N*_*G*_ > 2) and community stability was improved. Irrespective of system sizes, stability increases with increasing numbers of genotypes that can lead to differences in fitness (Fig 1). The resulting intraspecific variation has a major effect on stability, particularly in otherwise less stable complex systems. As the system becomes larger, stabilization due to intraspecific variation is sufficient to approach the stability of a simple system, indicating the stabilizing force of intraspecific variation (Fig 1). Hence, the powerful inherent instability associated with increases in system complexity can be greatly mitigated by intraspecific variations. These quantitative analyses were little affected when performed with different parameters (S1 Fig), with different stability indexes (S2 Fig), and with changing time scales of ecological and evolutionary dynamics (S3 Fig). As is obvious, the same result was also obtained in an alternative model with explicit population dynamics of genotypes (S4 Fig). Although network structures (random or cascade) do not affect the qualitative pattern of the positive effect of intraspecific variation to persistence, persistence tends to be lower in cascade model (Fig 1). In more complex systems with large values of connectance, the stability almost does not change with increasing intraspecific diversity or is not even destabilized (S5 Fig).

**Fig 2:**
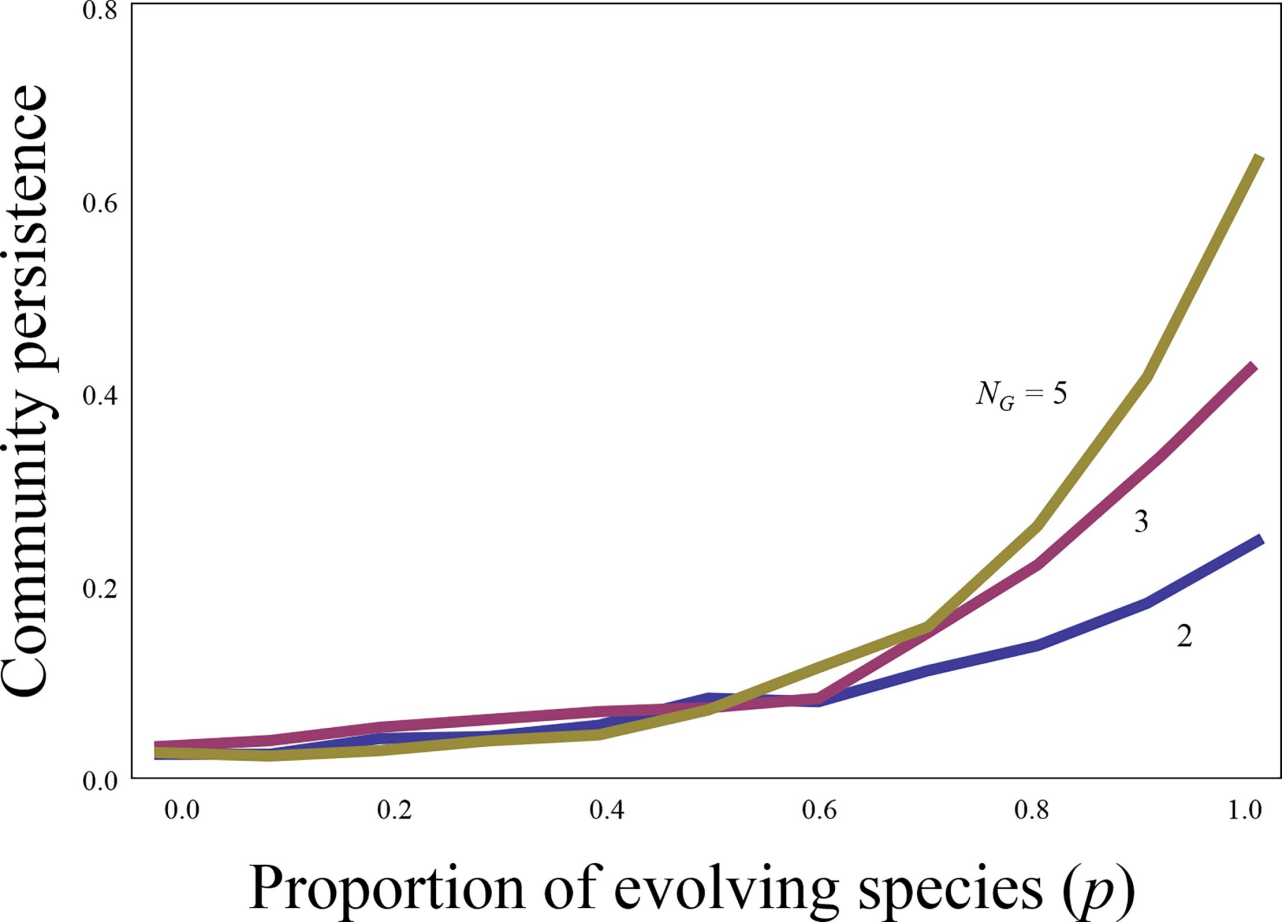
Effects of fractions of evolving species on community stability. The lines correspond with different numbers of genotypes (*N*_*G*_). *N*_*S*_ = 20 and *C =* 0.2.

The stabilizing effects of intraspecific variations in complex communities are contingent on two conditions, regardless of network structures. First, intraspecific variation needs to be present in multiple species. Consider a proportion (*p*) of evolving species with intraspecific variation; intraspecific variation fails to stabilize communities unless *p* is sufficiently large (Fig 2, S6 Fig). This is because evolving species can contribute to the persistence of communities. I examined which types of species become extinct and found that the extinct species are perfectly non-evolving species (i.e. evolving species are perfectly surviving) (S7 Fig). This clearly shows that intraspecific variation is necessary for the persistence of communities.

**Fig 3:**
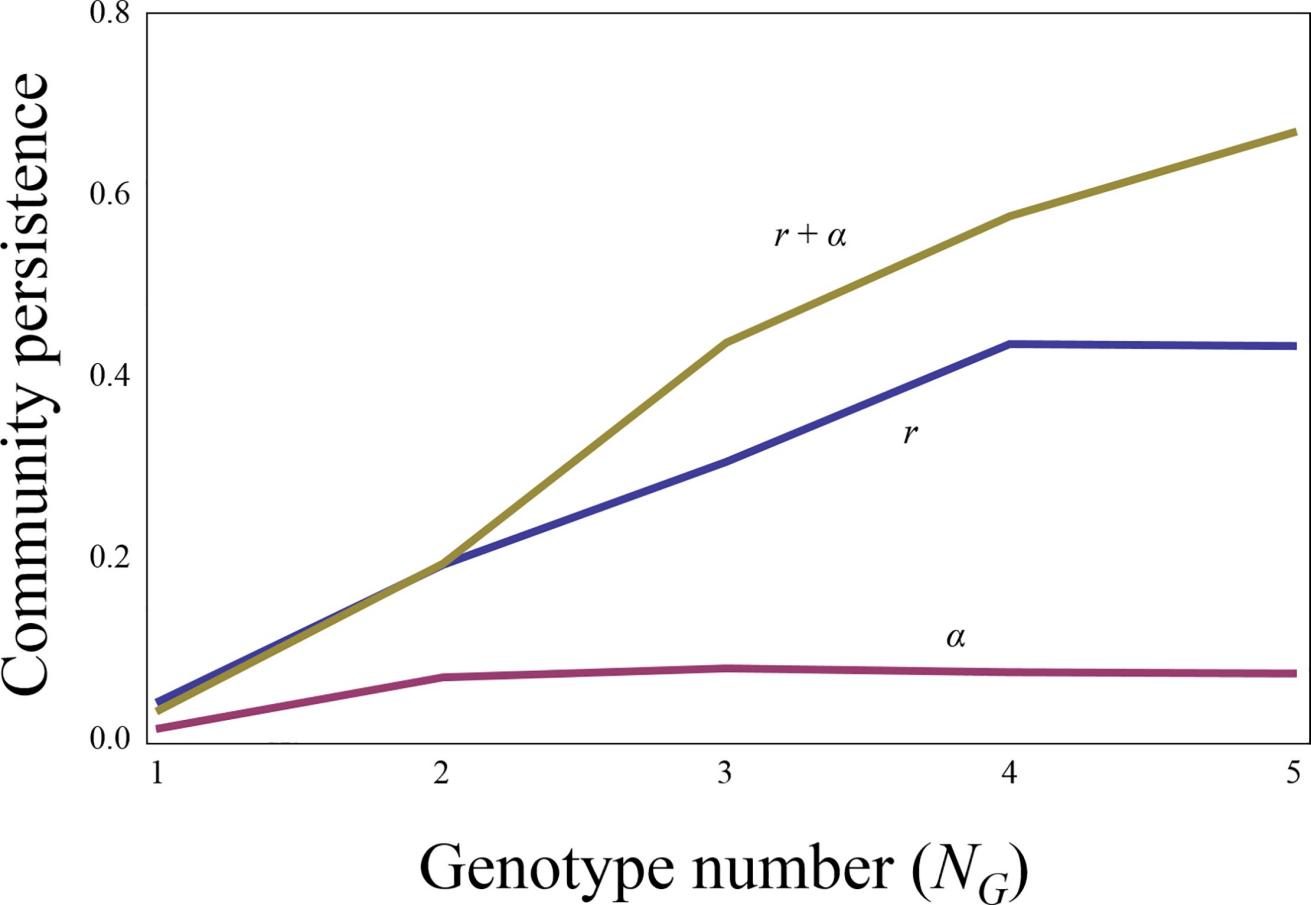
Types of evolving traits and community stability. The lines represent cases in which *α* and/or *r* evolve. *N*_*S*_ = 20 and *C =* 0.2.

Second, intraspecific variation is required in multiple traits. When intraspecific variation applies only to the strengths of interspecific interactions, coevolutionary cycles tend to fluctuate widely (S8 Fig). As a consequence, the stability of population dynamics is almost not improved, even with larger intraspecific variations (Fig 3, S9 Fig). In contrast, when intraspecific variations are applicable to growth rates only, rapid growers are selected (S8 Fig) and population sizes approach equilibrium, leading to increased stability (Fig 3, S9 Fig). However, variation in both traits leads to further increases in stability (Fig 3, S9 Fig). This synergistic effect was supported by a mathematical analysis in a simple food web comprising one predator and one prey, each of which had two genotypes (S1 Text). The analysis of feasibility and local stability in non-trivial equilibrium clearly showed that the variation in growth rates and interaction strengths between genotypes were necessary for stable coexistence.

In the above analysis, I assumed no discrimination between intra and interspecific variations of genotypes on parameters *r* and *α*, i.e., genotype of the same species can considerably differ and be similar to genotypes of different species. I relaxed this assumption by turning the parameter range of genotypes (genotype variability) and species. To consider this, first, I determined the species-specific parameter values (in *r* and *α*), which are randomly determined from the beta distribution β(*m*,*m*) with a parameter *m*. For convenience, I assumed *δ* = 1/*m*, where *δ* represents the level of interspecific variability.　As *δ* increases, variation increases. When *δ* = 1, it has the largest variation. Once determining mean values of each species, I calculated the values of shape parameters of gamma distribution γ(*k*,*θ*) in such a way that being equal to the species mean value and variance (which is the level of intraspecific variation (genotype variability) controlled by us). More specifically, I determined parameter values, *k* and *θ*, by calculating the simultaneous equations: “a species specific trait value = *kθ* (mean of gamma distribution)” and “genotype variability (*σ*) = *kθ*^2^ (variance of gamma distribution)”. After obtaining *k* and *θ* in each species, I randomly selected the trait values of each genotype from the species specific gamma distribution. By changing *σ* and *δ*, we can control the intra- and interspecific variabilities.

**Fig 4:**
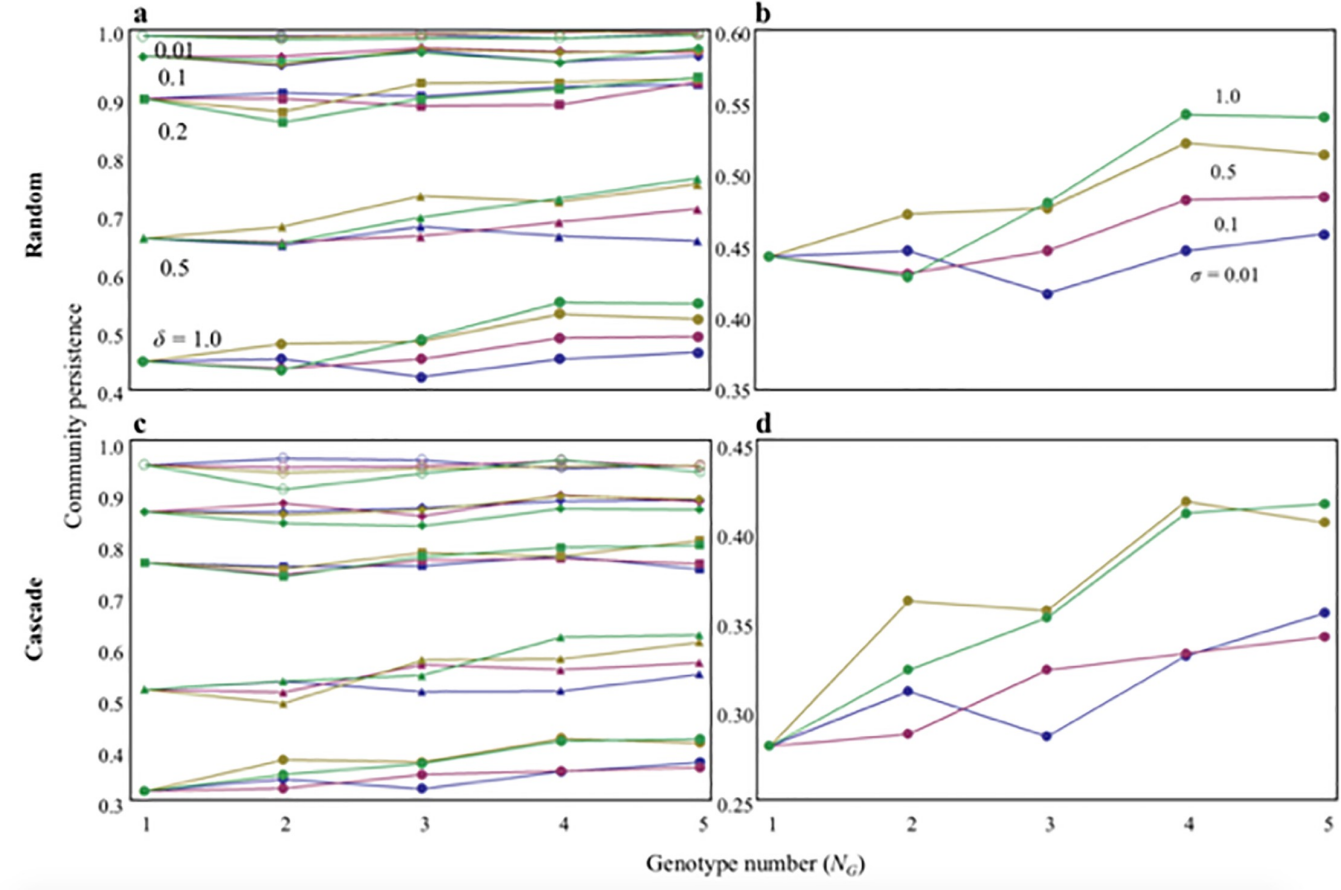
Effects of genotype variability on community stability. (a, b) random food web. (c, d) cascade food web. The mean values of *α* and *r* in each species were randomly determined using beta distribution. For analytical convenience, the each species specific mean value was multiplied by 10 and a very small value (0.011) was added. By using species specific mean parameter value and a given variance of the gamma distribution, the shape parameters of gamma distribution were determined. From the gamma distribution obtained, parameter values of each genotype in each species were randomly determined. Each parameter value was tuned by multiplying 0.05 so as to compare the main result (Fig 1). I assumed *N*_*S*_ = 10 and *C =* 0.2.

The analysis showed that with interspecific variation, persistence largely decreases when genotype variability is lower than the interspecific variability, regardless of the degree of variability, and is approximated to the result of a single genotype (Fig 4). The stabilizing effect owing to an increase in genotype number can appear if both genotype variability and interspecific variability are large. However, the stabilizing effect is not significant compared with a case without discrimination between genotype and interspecific variabilities (Figs 1 and 4). In this methodology, genotype variability cannot approach to the level of interspecific variation. Hence, it is not possible to show the significant stabilizing effect owing to an increase in genotype number. Conversely, without interspecific variation, persistence is almost unaffected by genotype number because the stability is already high (Fig 4). These results suggest that the two components of intraspecific diversity, variability and number of genotypes, play some roles in community persistence, particularly when the system has interspecific variation.

## Discussion

Heritable intraspecific variation allows adaptive evolution by natural selection in response to ecological interactions, and in turn alters ecological interactions [14]. The present model indicates that these eco-evolutionary dynamics may stabilize otherwise less stable complex communities. Particularly, stabilization is likely to occur in less connected food webs. For evolution to stabilize community dynamics, this model suggests that greater intraspecific variation is necessary in many species as well as in multiple traits. Furthermore, variability and number of genotypes play a role in stabilizing the communities. The significant stabilizing effect is likely to be observed particularly when intraspecific variation is as large as interspecific variation.

The instability that is inherent in systems with species diversity may be mitigated by intraspecific diversity. Because species-rich complex systems are complex environments for all of their member species, more variation may be required to improve conditions or express adaptive traits. In the present model framework, new adaptive traits cannot occur because the model does not accommodate mutations. Hence, natural mutation–selection processes that produce more adaptive traits may further stabilize complex systems. Under these conditions, evolution may continue to stabilize communities in a self-organized manner, warranting maintenance of intraspecific variation in all species. Hence, in complex environments, interspecific diversity may be a key selective force that maintains intraspecific variation [51]. Accordingly, the present model indicates that intraspecific variation is more likely to be maintained in more complex systems, despite being limited by selection (S10 Fig). Perhaps intraspecific and interspecific diversity support each other during evolution [32]. Conversely, even with intraspecific diversity, more complex systems are less stable than simple systems. It comes from an inherent effect due to interaction strength variation [52], suggesting that complex systems have qualitatively different rules than simple systems.

The roles of intraspecific diversity in multispecies community dynamics have predominantly been examined in competition model systems. In one such study, competition communities with clonal genotypes were simulated to show that within-species trait diversity promotes multispecies coexistence, but intra and interspecific variations were not considered in this model [53]. Exchanges of genetic material between individuals also calculably promoted species diversity under environmental fluctuations [54]. Another recent quantitative genetics model shows that only heritable fixed intraspecific variations contributed considerable resilience to communities suffering environmental disturbances [55]. Furthermore, a food web model showed that faster evolutionary changes in traits promoted species coexistence, although again intraspecific variation was not explicitly considered [56]. These studies support the present theory and suggest a general stabilizing role of intraspecific diversity in community dynamics across various systems with different interaction types. Furthermore, empirical studies showed a tendency toward positive links between intraspecific genetic diversity and species diversity [32]. However, almost all of the previous model systems are competitive and are of a single trophic level. Further studies are thus required to test the present prediction in broad systems, including food webs with multiple trophic levels.

The present computations suggest that intraspecific variations in multiple traits play key roles in stabilizing systems. Yet, alone, variations in interspecific interaction strength offer limited stabilizing effects on community dynamics. Although evolution of species interactions improves the fitness of the involved species, it causes Red queen coevolutionary cycles between species [57,58], albeit with a stable median state amid fluctuating cycles of selection. Under these conditions, inherently slow growing species remain unlikely to persist, resulting in decreased stability of the community. But because evolution of growth rates follows selection of the fastest growing genotype, it improves species fitness and the disadvantages for species interactions remain unchanged. Therefore, by mutual compensation, variations in both traits can improve fitness, thereby facilitating persistence of each species. Diversity of multiple traits may also be important for adaptation to diverse environments, although the roles of genetic architectures and correlations between multiple traits in community dynamics remain subjects of future studies.

The present results show that significant stabilizing effect due to natural selection needs a large intraspecific variation comparable to interspecific variation. In other words, it suggests that the contribution of natural selection for community stability is not much large if the intraspecific variation is not sufficiently high. It does not seem intuitive because one may think that interspecific variation is much larger than intraspecific variation in nature. On the contrary, it also suggests that intraspecific variation may be much larger than expected. Although it needs systematic comparison between intra- and interspecific variations in natural communities, such efforts are insufficient to test the present model prediction. Even if the intraspecific variation is found to be much lower than interspecific variation in nature, the intraspecific variation, although high in the past, may be reduced by evolution and maintaining community stability. In addition, even if the intraspecific variation is not so large, occasional large mutation or gene flow from external systems may play a key role in broadening intraspecific variation. A recent study demonstrated that ecological effects of intraspecific variation are comparable to, and sometimes stronger than, species effects [59]. In addition to such studies, revealing the real patterns of intraspecific and interspecific variations and the changes in intraspecific variations over time will confirm the validity of the present model.

Biodiversity, including intra and interspecific diversities, may be self-sustaining having implications for the conservation of biodiversity. Environmental destruction from overexploitation, species loss, and habitat fragmentation, may decrease the stabilizing power of ecosystems. Overexploitation has been shown to reduce intraspecific diversity [60] and can potentially threaten even highly adaptive individuals [61,62], in part, by weakening the effects of natural selection. Species loss reduces the complexity of ecological communities favoring maintenance of intraspecific variation. Habitat fragmentation not only reduces intraspecific diversity within local communities but also eliminates gene flows, which are an important source of genetic variations [63,64]. Finally, loss of intraspecific diversity in only a few species may significantly impair the maintenance of stability in the entire ecosystem.

## Supporting information

S1 Text Mathematical analysis. (DOCX)

S1 Code Mathematica codes for producing figures. (NB)

S1 FigParameter dependence of the effects of intraspecific variation on community stability.(a) Random food web. (b) Cascade food web. The lines represent sets of parameters ($\bar{\alpha }$, $\bar{r}$). *N*_*S*_ = 20 and *C =* 0.2. Other information is the same as that of Fig 1.(PDF)

S2 Fig Effects of intra- and interspecific diversity on stability. Stability is evaluated as the mean number of species that survive. The error bar represents the standard deviation. (a) Random food web (b) Cascade food web. Other information is the same as that of Fig 1.
(PDF)

S3 FigEffects of the speed of evolution on the relationship between intraspecific variation and stability.The difference between the time scales of ecological and evolutionary dynamics is described by extending Eq (4) (in the text) to d*f*_*ij*_/dt = $Gf_{ij}(w_{ij}-{\bar{w}}_{i}),$ where *G* is the speed of evolution. Red, green, blue, and black dashed lines represent different values of *G*—0.05, 0.1, 0.2, and 1, respectively. Other information is the same as that of Fig 1A.(PDF)

S4 Fig Effects of intra- and interspecific diversity on stability in an alternative model with explicit population dynamics of genotypes. The model is described in S1 Text. Whether a species goes extinct was evaluated by the total population size of the genotypes in the species. (a) Random food web (b) Cascade food web. Other information is the same as that of Fig 1.
(PDF)

S5 Fig Effects of connectance on community stability. (a) Random food web. *N*_*S*_ = 20. (b) Cascade food web. *N*_*S*_ = 15. Different colors represent different values of connectance. Other information is the same as that of Fig 1.
(PDF)

S6 Fig Effects of fractions of evolving species on community stability in cascade food web. The lines correspond with different numbers of genotypes (*N*_*G*_). *N*_*S*_ = 10 and *C =* 0.2.
(PDF)

S7 Fig Contributions of evolving and non-evolving species for community persistence. I considered two types of species with intraspecific variation (*N*_*G*_ = 2) or without variation. I determined which species did not survive and calculated the probability that the extinct species is non-evolving species. *N*_*S*_ = 20 and *C =* 0.2. Other information is the same as that of Fig 1A.
(PDF)

S8 Fig Proportion of persistent communities with intraspecific variation. (a) Random food web. (b) Cascade food web. The lines represent types of evolving traits (see Fig 4). I calculated the proportion of persistent communities in which either of the species has > 2 genotypes. If the frequency of a genotype in a species after reaching the final time step is >1−10^−5^, the focal species is considered to have a single genotype. Otherwise, the focal species is considered to have > 2 genotypes at least. Then, if either of the species has > 2 genotypes, the focal persistent community is considered to maintain intraspecific variation within a community. *N*_*S*_ = 20 and *C =* 0.2.
(PDF)

S9 Fig Types of evolving traits and community stability in cascade food web. The lines represent cases in which *α* and/or *r* evolve. *N*_*S*_ = 20 and *C =* 0.2. Other information is the same as that of Fig 1.
(PDF)

S10 Fig Proportion of persistent communities with intraspecific variation, varying with differing species richness. (a) Random food web. (b) Cascade food web. The lines represent species richness. Other details are as described for S4 Fig.
(PDF)

S11 Fig Relationship between the consumption rates and local stability of the equilibrium in one predator–one prey with two genotype systems. In blue and gray regions, the equilibrium is locally stable and unstable, respectively. In unstable cases, a limit cycle occurs (S12 Fig). In white regions, the equilibrium is trivial and coexistence cannot occur. Parameter values *r*_*ij*_ are varied in each panel. (a) *r*_11_ = 1.0, *r*_12_ = 0.8, *r*_21_ = *r*_22_ = 0.1. (b) *r*_11_ = *r*_12_ = 1.0, *r*_21_ = *r*_22_ = 0.1. (c) *r*_11_ = 1.0, *r*_12_ = 1.2, *r*_21_ = *r*_22_ = 0.1. (d) *r*_11_ = *r*_12_ = 1.0, *r*_21_ = 0.1, *r*_22_ = 0.05. (e) *r*_11_ = *r*_12_ = 1.0, *r*_21_ = 0.1, *r*_22_ = 0.15. (f) *r*_11_ = 1.0, *r*_12_ = 0.8, *r*_21_ = 0.1, *r*_22_ = 0.15. Other parameter values are: *g* = 0.5, *a*_21_ = 1.0, and *a*_22_ = 0.1.
(PDF)

S12 Fig Examples of population and genotype dynamics in one predator–one prey with two genotypes. (a, b) Unstable system with a limit cycle. I assumed *r*_11_ = *r*_12_ = 1.0. (c, d) Stable system. I assumed *r*_11_ = 1.0 and *r*_12_ = 0.8. Small panels in (a) and (b) are the phase plots of population dynamics and genotype dynamics, respectively, after a sufficiently long period (from 39000 to 40000 time steps). The ranges of horizontal (h) and vertical (v) axes in (a) and (b) are (h: 0.82070–0.821 and v: 0.32565–0.32582) and (h: 0.47–0.53 and v: 0.47–0.53), respectively. Other parameter values are *r*_21_ = *r*_22_ = 0.1, *g* = 0.5, *a*_21_ = 1.0, and *a*_22_ = 0.1.
(PDF)
