# Supplementary material for: Natural selection contributes to food web stability
Source: PLoS One. 2020 Jan 10;15(1):e0227420. doi: 10.1371/journal.pone.0227420 (PMC6953789; doi:10.1371/journal.pone.0227420)
Supplement: S1 Text — (DOCX) [file pone.0227420.s001.docx]

**Supplementary Information**

**Appendix A**

***The simplest food-web model***

Consider a one-predator-one prey system with two genotypes based on the model framework in the main text. In this framework, note that predator species is either autotrophs or use external resources. Then, the fitness of genotypes for each species is represented as follows:

$w_{11}=r_{11} -X_{1} -a_{1121}f_{21}X_{2} -a_{1122}f_{22}X_{2} ,$ (S-1a)

$w_{12}=r_{12} -X_{1} -a_{1221}f_{21}X_{2} -a_{1222}f_{22}X_{2} ,$ (S-1b)

$w_{21}=r_{21} -X_{2} +g\left(a_{1121}f_{11}X_{1} +a_{1221}f_{12}X_{1} \right),$ (S-1c)

$w_{22}=r_{22} -X_{2} +g\left(a_{1122}f_{11}X_{1} +a_{1222}f_{12}X_{1} \right),$ (S-1d)

where *w*_11_ and *w*_12_ represent the fitness of genotype 1 and 2 for prey species 1, and *w*_21_ and *w*_22_ represent the fitness of genotype 1 and 2 for predator species 2, respectively. Note that the strength of self regulation is 1 as with the full model in the main text. For the simplicity, the notations are replaced as follows: *a*_1121_ = *a*_11_, *a*_1122_ = *a*_21_, *a*_1221_ = *a*_12_, and *a*_1222_ = *a*_22_. Based on these fitness equations and differential equations (1) and (4) in the main text, we can analyse the predator-prey system with two genotypes.

***Feasibility and stability of equilibrium***

The equilibrium is obtained by setting the differential equations defined in the above to zero. Then we have the following equilibrium:

$X_{1}^{*}=\frac{-r_{22}+f_{21}^{*}\left(r_{22}-r_{21}\right)+X_{2}^{*}}{\left[a_{22}f_{12}^{*}f_{22}^{*}+a_{12}f_{21}^{*}+f_{11}^{*}\left\{a_{21}f_{22}^{*}+\left(a_{11}-a_{12}\right)f_{11}^{*}\right\}\right]g},$ (S-2a)

$X_{2}^{*}=\frac{r_{12}+f_{11}^{*}\left(r_{11}-r_{12}\right)-X_{1}^{*}}{a_{22}f_{12}^{*}f_{22}^{*}+a_{12}f_{21}^{*}+f_{11}^{*}\left\{a_{21}f_{22}^{*}+\left(a_{11}-a_{12}\right)f_{11}^{*}\right\}},$ (S-2b)

$f_{11}^{*}=\frac{-r_{21}+r_{22}+\left(-a_{12}+a_{22}\right)gX_{1}^{*}}{\left(a_{11}-a_{12}-a_{21}+a_{22}\right)gX_{1}^{*}},$ (S-2c)

$f_{21}^{*}=\frac{r_{11}-r_{12}+\left(-a_{21}+a_{22}\right)X_{2}^{*}}{\left(a_{11}-a_{12}-a_{21}+a_{22}\right)X_{2}^{*}},$ (S-2d)

where asterisk represents the equilibrium.

Here, I examine the effects of intraspecific variation in *a_ij_* and *r_ij_*. First, without variation between genotypes in *a_ij_* (i.e. *a*_11_ = *a*_21_ and *a*_12_ = *a*_22_), it intuitively does not make the equilibrium feasible or has not non-trivial equilibrium (denominators in equations (S-2) becomes zero). Next, without variation between genotypes in *r_ij_* (i.e. *r*_11_ = *r*_12_ = *r*_1_ and *r*_21_ = *r*_22_ = *r*_2_), the equilibrium becomes:

$X_{1}^{*}=\frac{\left(a_{11}-a_{12}-a_{21}+a_{22}\right)\left\{\left(a_{11}-a_{12}-a_{21}+a_{22}\right)r_{1}+\left(a_{12}a_{21}-a_{11}a_{22}\right)r_{2}\right\}}{\left(a_{11}-a_{12}-a_{21}+a_{22}\right)^{2}+\left(a_{12}a_{21}-a_{11}a_{22}\right)^{2}g},$ (S-3a)

$X_{2}^{*}=\frac{\left(a_{11}-a_{12}-a_{21}+a_{22}\right)\left\{\left(a_{11}-a_{12}-a_{21}+a_{22}\right)r_{2}-\left(a_{12}a_{21}-a_{11}a_{22}\right)gr_{1}\right\}}{\left(a_{11}-a_{12}-a_{21}+a_{22}\right)^{2}+\left(a_{12}a_{21}-a_{11}a_{22}\right)^{2}g},$ (S-3b)

$f_{11}^{*}=\frac{a_{22}-a_{12}}{a_{11}-a_{12}-a_{21}+a_{22}},$ (S-3c)

$f_{21}^{*}=\frac{a_{22}-a_{21}}{a_{11}-a_{12}-a_{21}+a_{22}},$ (S-3d)

For the analytical simplicity, I consider a special case where predation rates of two predators to different prey genotypes are same (i.e. *a*_12_ = *a*_21_ = *a* and *a*_11_ = *a*_22_ = *a*’). Here, I assume *a* > *a*’ (the argument is essentially same even if the condition is reversed). Then, the feasible condition of the non-trivial equilibrium is as below,

*r*_1_/*r*_2_ > (*a* + *a*’)/2 (S-4)

This suggests that even with variation in interaction strengths between genotypes, interspecific variation in growth rates can make the equilibrium infeasible.

Even if the equilibrium is feasible, they may not stably coexist, because the equilibrium might not be stable. Thus, here, using a local stability analysis, I examine the stability of the coexistence equilibrium. The local stability of the system described by the above was performed by linearizing the dynamics near the nontrivial equilibrium. Stability was judged by whether the characteristic equation of their Jacobian matrix satisfied the Routh–Hurwitz criteria. The Jacobian matrix was calculated under the equilibrium,

$$J=\left(\begin{matrix} \frac{-4r_{1}+2r_{2}\left(a+a'\right)}{4+g\left(a+a'\right)^{2}} & \frac{\left(a+a'\right)\left(-2r_{1}+r_{2}\left(a+a'\right)\right)}{4+g\left(a+a'\right)^{2}} & 0 & 0 \\ \frac{g\left(a+a'\right)\left(2r_{2}+gr_{1}\left(a+a'\right)\right)}{4+g\left(a+a'\right)^{2}} & -\frac{2\left(2r_{2}+gr_{1}\left(a+a'\right)\right)}{4+g\left(a+a'\right)^{2}} & 0 & 0 \\ 0 & 0 & 0 & -\frac{\left(a'-a\right)\left(2r_{2}+gr_{1}\left(a+a'\right)\right)}{4+g\left(a+a'\right)^{2}} \\ 0 & 0 & -\frac{g\left(a'-a\right)\left(-2r_{1}+r_{2}\left(a+a'\right)\right)}{4+g\left(a+a'\right)^{2}} & 0 \end{matrix}\right)$$

The characteristic equation for determining the eigenvalues is$\lambda^{4}+w_{1}\lambda^{3}+w_{2}\lambda^{2}+w_{3}\lambda+w_{4}=0.$The equilibrium point is locally stable if *w*_1_, *w*_3_, *w*_4_ > 0 and $w_{1}\left(w_{2}w_{3}-w_{1}w_{4}\right)-w_{3}^{2}>0,$ according to the Routh–Hurwitz criteria. I obtain the coefficients of characteristic equation, *w_i_* (*i* =1,..., 4). Then, I found that a necessary condition for stability, $w_{1}\left(w_{2}w_{3}-w_{1}w_{4}\right)-w_{3}^{2}>0$, is always not held ($w_{1}\left(w_{2}w_{3}-w_{1}w_{4}\right)-w_{3}^{2}=0$). Hence, in this special case, the equilibrium is always unstable. This suggests that the stable coexistence is not possible even with variation in interaction strengths between genotypes or the predators prefer to utilize different prey genotypes.

Full model analysis performed by numerically calculating eigenvalues of Jacobian matrix supports this mathematical result. Figure S11 shows cases where the above strong assumptions are relaxed. For stable coexistence, the variations in growth rates and interaction strengths between genotypes are required.

**Appendix B**

***Consistency with the explicit genotype dynamics model***

I can show that the present model is exactly same with explicit genotype dynamics model as below.

First, I obtain mean fitness $\bar{w}_{i}=\frac{dX_{i} /dt}{X_{i} }$ by deforming the equation (1) in the text. By substituting the mean fitness into the equation (4) in the text, I have

$\frac{df_{ij}}{dt}=f_{ij}(w_{ij}-\frac{dX_{i}/dt}{X_{i} }),$ (S-5)

By multiplying both sides of the above equation (S-5) by *X_i_*, I have

$\frac{df_{ij}}{dt}\cdot X_{i} =f_{ij}X_{i} w_{ij}-f_{ij}\frac{dX_{i}}{dt},$ (S-6)

By moving the second term in the right hand side of (S-6) to the left hand side, I have

$\frac{df_{ij}}{dt}\cdot X_{i} +\frac{dX_{i}}{dt}f_{ij}=f_{ij}X_{i} w_{ij}$ (S-7)

Since the left hand side of (S-7) equals to d*f_ij_X_i_*/dt, equation (S-7) becomes to

$\frac{df_{ij}X_{i}}{dt}=f_{ij}X_{i}w_{ij}$ (S-8)

By defining *f_ij_X_i_* = *X_ij_* (where *X_ij_* is the population size of each genotype *j* in a focal species *i*), finally I have

$\frac{dX_{ij}}{dt}=X_{ij}w_{ij}$ (S-9)
