# Supplementary figures and images for: Natural selection contributes to food web stability
Source: PLoS One. 2020 Jan 10;15(1):e0227420. doi: 10.1371/journal.pone.0227420 (PMC6953789; doi:10.1371/journal.pone.0227420)

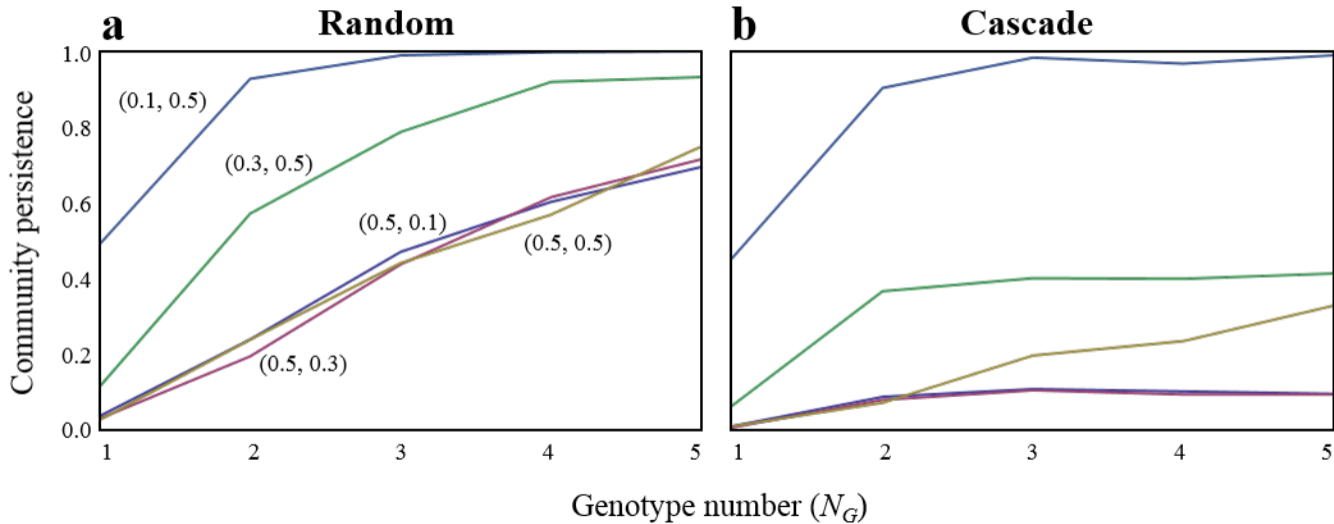

Supplement: S1 Fig — (a) Random food web. (b) Cascade food web. The lines represent sets of parameters (α¯, r¯). NS = 20 and C = 0.2. Other information is the same as that of Fig 1. (PDF) [file pone.0227420.s003.pdf]

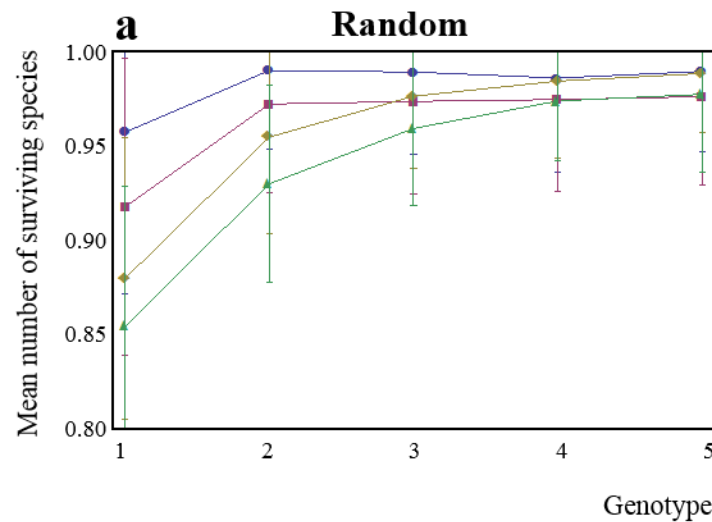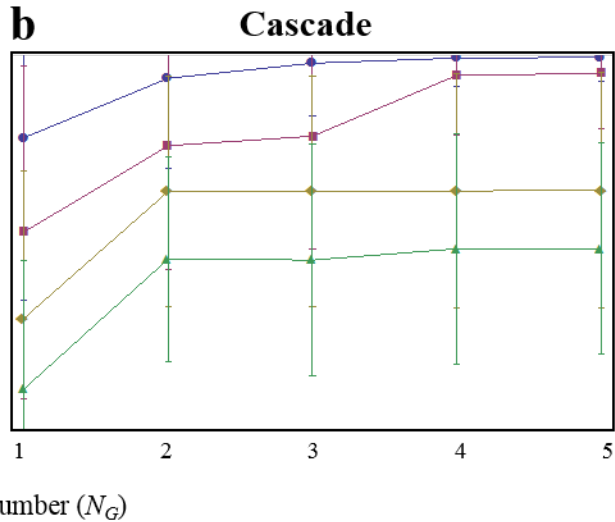

Supplement: S2 Fig — Stability is evaluated as the mean number of species that survive. The error bar represents the standard deviation. (a) Random food web (b) Cascade food web. Other information is the same as that of Fig 1. (PDF) [file pone.0227420.s004.pdf]

Community persistence

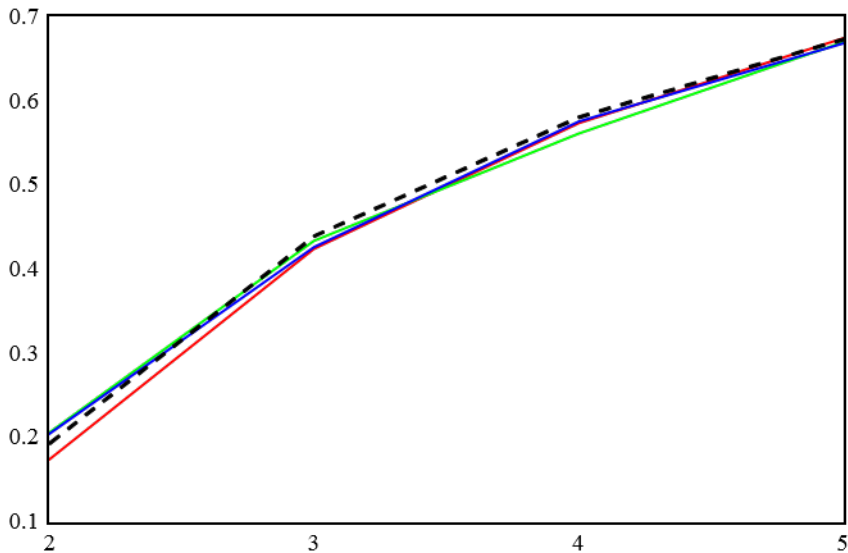

Genotype number ( $N_G$ )

Supplement: S3 Fig — The difference between the time scales of ecological and evolutionary dynamics is described by extending Eq (4) (in the text) to dfij/dt = Gfij(wij−w¯i), where G is the speed of evolution. Red, green, blue, and black dashed lines represent different values of G—0.05, 0.1, 0.2, and 1, respectively. Other information is the same as that of Fig 1A. (PDF) [file pone.0227420.s005.pdf]

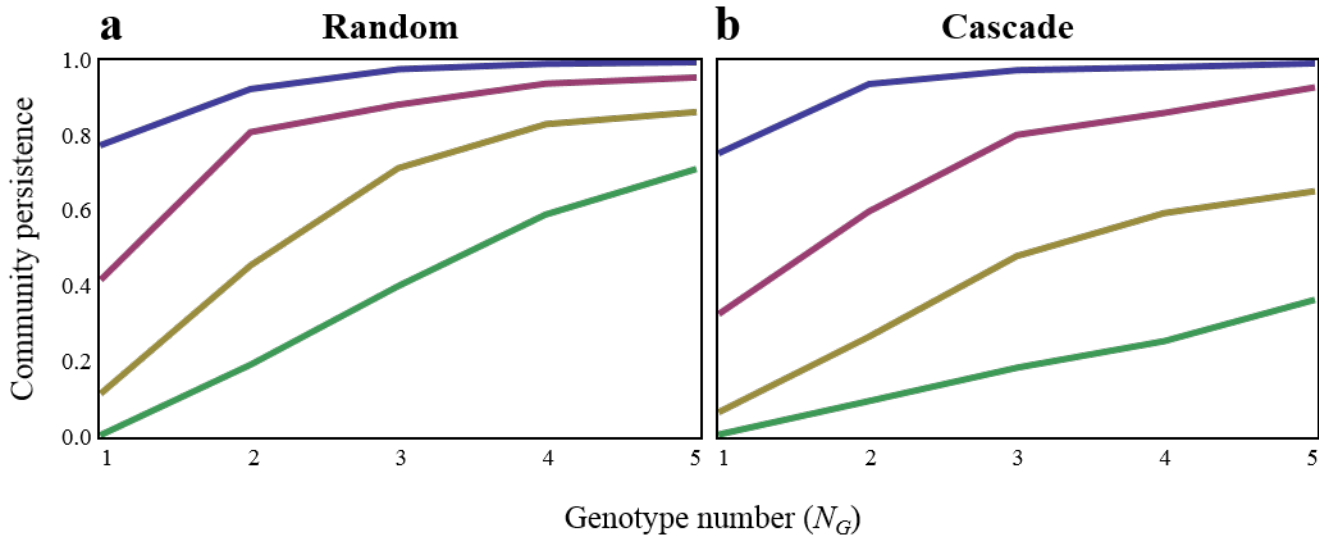

Supplement: S4 Fig — The model is described in S1 Text. Whether a species goes extinct was evaluated by the total population size of the genotypes in the species. (a) Random food web (b) Cascade food web. Other information is the same as that of Fig 1. (PDF) [file pone.0227420.s006.pdf]

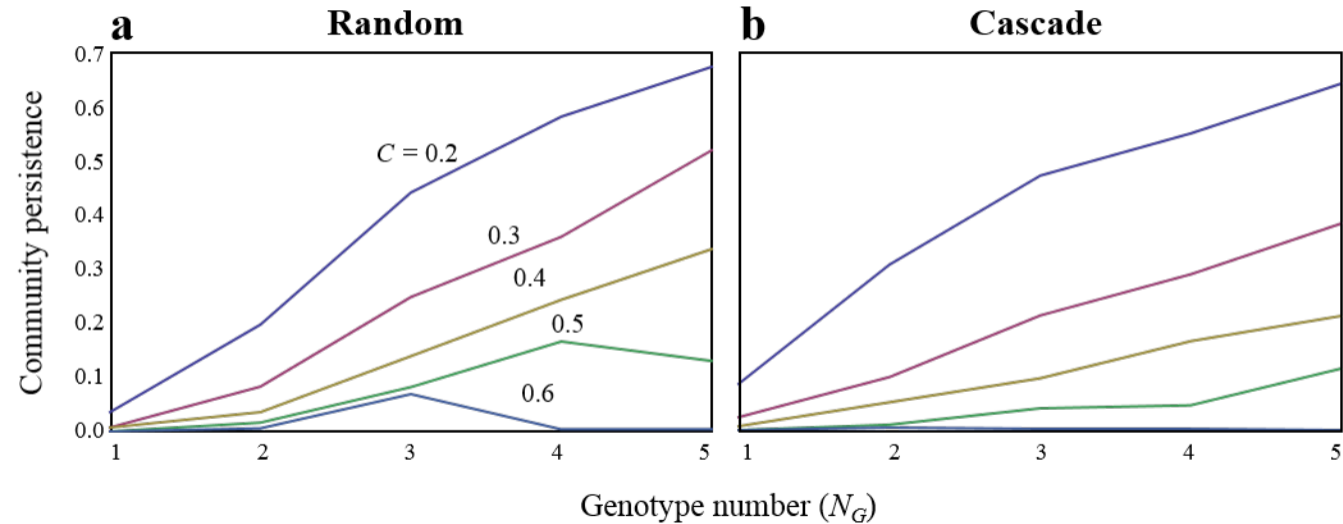

Supplement: S5 Fig — (a) Random food web. NS = 20. (b) Cascade food web. NS = 15. Different colors represent different values of connectance. Other information is the same as that of Fig 1. (PDF) [file pone.0227420.s007.pdf]

Community persistence

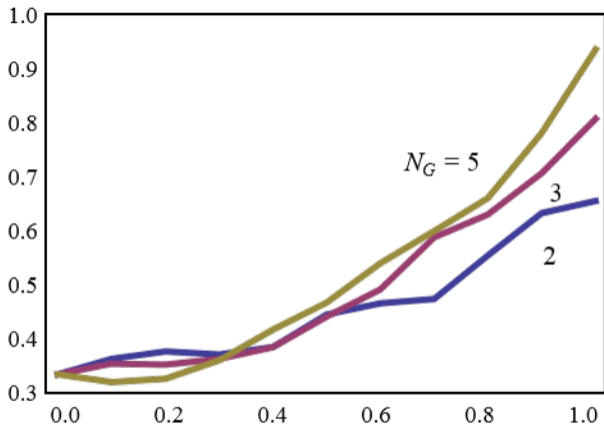

Proportion of evolving species ( $p$ )

Supplement: S6 Fig — The lines correspond with different numbers of genotypes (NG). NS = 10 and C = 0.2. (PDF) [file pone.0227420.s008.pdf]

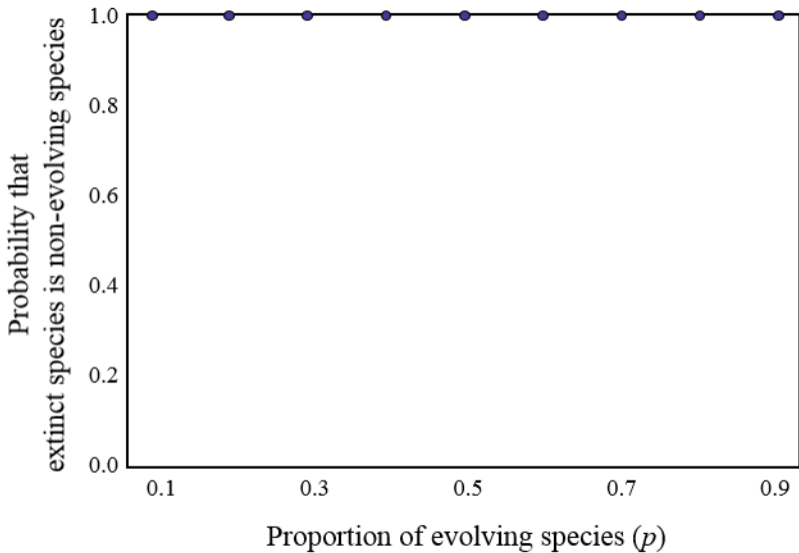

Supplement: S7 Fig — I considered two types of species with intraspecific variation (NG = 2) or without variation. I determined which species did not survive and calculated the probability that the extinct species is non-evolving species. NS = 20 and C = 0.2. Other information is the same as that of Fig 1A. (PDF) [file pone.0227420.s009.pdf]

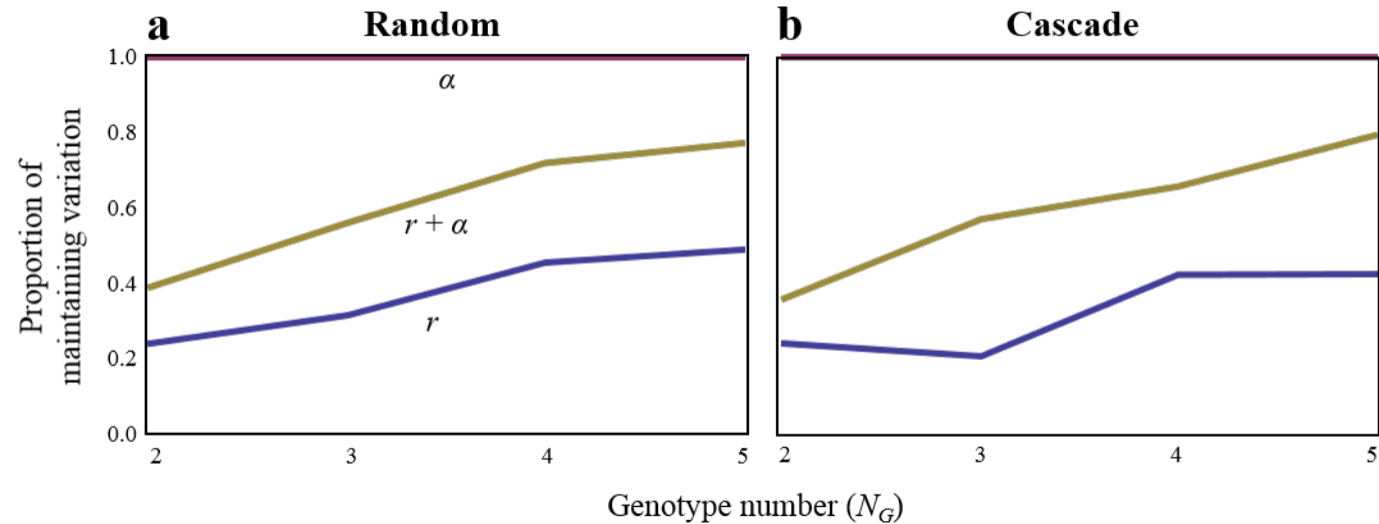

Supplement: S8 Fig — (a) Random food web. (b) Cascade food web. The lines represent types of evolving traits (see Fig 4). I calculated the proportion of persistent communities in which either of the species has > 2 genotypes. If the frequency of a genotype in a species after reaching the final time step is >1−10−5, the focal species is considered to have a single genotype. Otherwise, the focal species is considered to have > 2 genotypes at least. Then, if either of the species has > 2 genotypes, the focal persistent community is considered to maintain intraspecific variation within a community. NS = 20 and C = 0.2. (PDF) [file pone.0227420.s010.pdf]

Community persistence

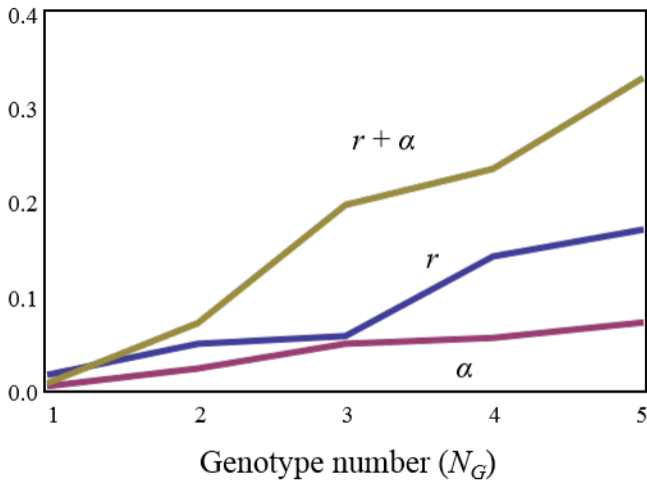

Supplement: S9 Fig — The lines represent cases in which α and/or r evolve. NS = 20 and C = 0.2. Other information is the same as that of Fig 1. (PDF) [file pone.0227420.s011.pdf]

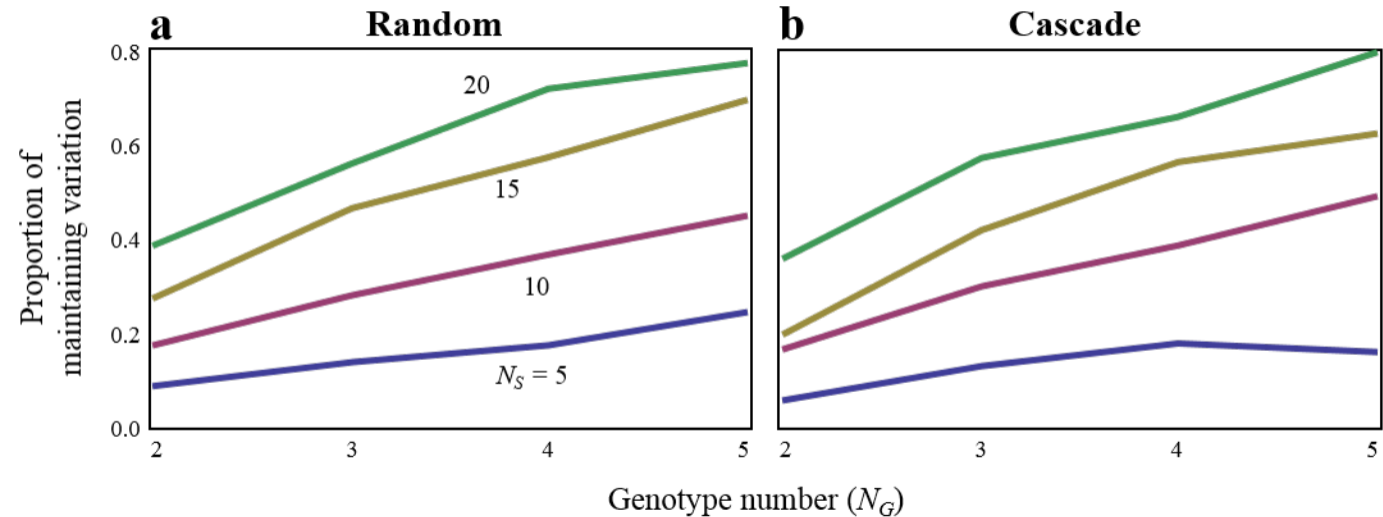

Supplement: S10 Fig — (a) Random food web. (b) Cascade food web. The lines represent species richness. Other details are as described for S4 Fig. (PDF) [file pone.0227420.s012.pdf]

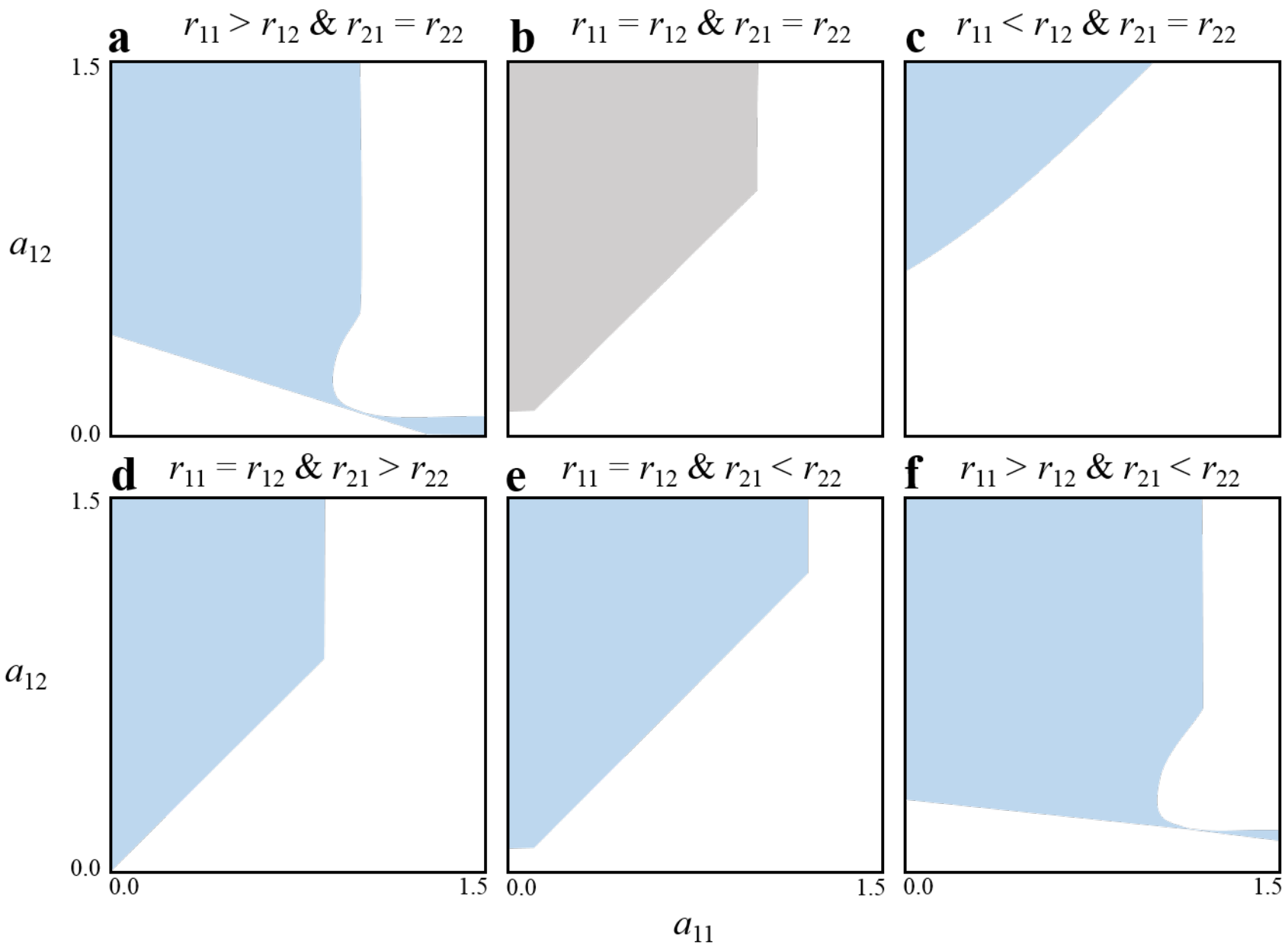

Supplement: S11 Fig — In blue and gray regions, the equilibrium is locally stable and unstable, respectively. In unstable cases, a limit cycle occurs (S12 Fig). In white regions, the equilibrium is trivial and coexistence cannot occur. Parameter values rij are varied in each panel. (a) r11 = 1.0, r12 = 0.8, r21 = r22 = 0.1. (b) r11 = r12 = 1.0, r21 = r22 = 0.1. (c) r11 = 1.0, r12 = 1.2, r21 = r22 = 0.1. (d) r11 = r12 = 1.0, r21 = 0.1, r22 = 0.05. (e) r11 = r12 = 1.0, r21 = 0.1, r22 = 0.15. (f) r11 = 1.0, r12 = 0.8, r21 = 0.1, r22 = 0.15. Other parameter values are: g = 0.5, a21 = 1.0, and a22 = 0.1. (PDF) [file pone.0227420.s013.pdf]

**a**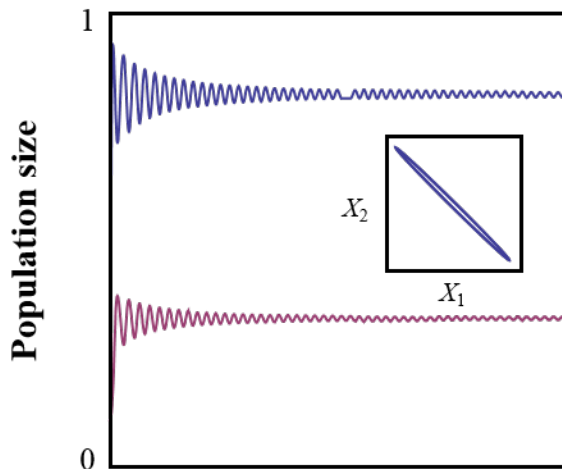**c**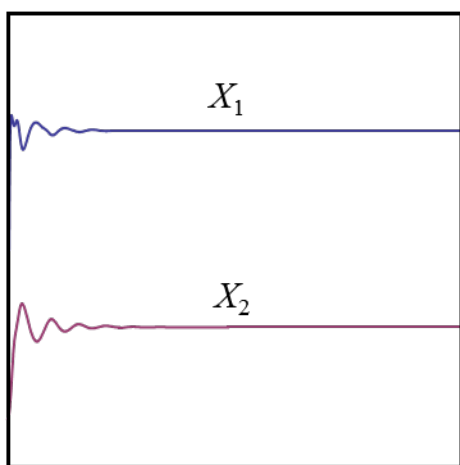**b**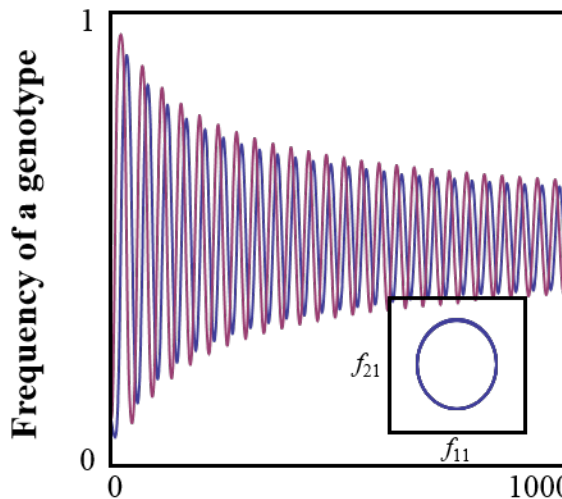**d**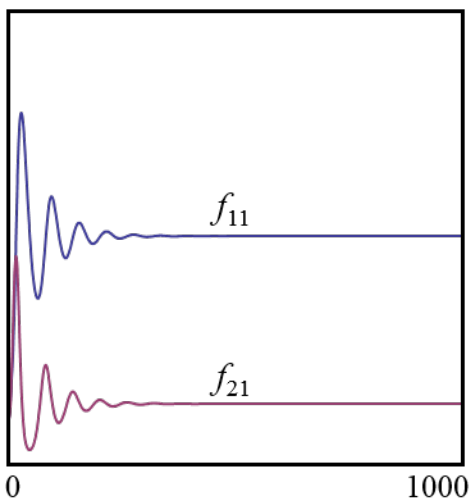

time

Supplement: S12 Fig — (a, b) Unstable system with a limit cycle. I assumed r11 = r12 = 1.0. (c, d) Stable system. I assumed r11 = 1.0 and r12 = 0.8. Small panels in (a) and (b) are the phase plots of population dynamics and genotype dynamics, respectively, after a sufficiently long period (from 39000 to 40000 time steps). The ranges of horizontal (h) and vertical (v) axes in (a) and (b) are (h: 0.82070–0.821 and v: 0.32565–0.32582) and (h: 0.47–0.53 and v: 0.47–0.53), respectively. Other parameter values are r21 = r22 = 0.1, g = 0.5, a21 = 1.0, and a22 = 0.1. (PDF) [file pone.0227420.s014.pdf]
